# Time from first tumor manifestation to diagnosis in patients with GEP-NET: Results from a large German tertiary referral center

**DOI:** 10.1097/MD.0000000000027276

**Published:** 2021-09-17

**Authors:** Christine Koch, Esra Koca, Natalie Filmann, Gabriele Husmann, Jörg Bojunga

**Affiliations:** a Goethe University Frankfurt, University Hospital, Department of Gastroenterology, Hepatology and Endocrinology, Frankfurt, Germany; b Goethe University Frankfurt, Department of Biostatistics and Mathematical Modeling, Frankfurt, Germany; c Goethe University Frankfurt, University Hospital, University Cancer Center, Tumor Documentation, Frankfurt, Germany.

**Keywords:** delay, diagnosis, metastases, neuroendocrine

## Abstract

Patients with neuroendocrine tumors (NET) often go through a long phase between onset of symptoms and initial diagnosis.

Assessment of time to diagnosis and pre-clinical pathway in patients with gastroenteropancreatic NET (GEP-NET) with regard to metastases and symptoms.

Retrospective analysis of patients with GEP-NET at a tertiary referral center from 1984 to 2019; inclusion criteria: Patients ≥18 years, diagnosis of GEP-NET; statistical analysis using non-parametrical methods.

Four hundred eighty-six patients with 488 tumors were identified; median age at first diagnosis (478/486, 8 unknown) was 59 years; 52.9% male patients. Pancreatic NET: 143/488 tumors; 29.3%; small intestinal NET: 145/488 tumors, 29.7%. 128/303 patients (42.2%) showed NET specific and 122/486 (25%) patients other tumor-specific symptoms. 222/279 patients had distant metastases at initial diagnosis (187/222 liver metastases). 154/488 (31.6%) of GEP-NET were incidental findings. Median time from tumor manifestation (e.g., symptoms related to NET) to initial diagnosis across all entities was 19.5 (95% CI: 12–28) days. No significant difference in patients with or without distant metastases (median 73 vs 105 days, *P* = .42).

A large proportion of GEP-NET are incidental findings and only about half of all patients are symptomatic at the time of diagnosis. We did not find a significant influence of the presence of metastases on time to diagnosis, which shows a large variability with a median of <30 days.

## 1 Introduction

Neuroendocrine tumors (NET) are a group of rare and heterogenic tumors that can arise in different organs throughout the body, predominantly in lung, pancreas, and small intestine.^[1,2]^ Overall incidence in Europe is 2.5/100,000 per year,^[2]^ making it an orphan disease.^[3]^ NET occurring in stomach, small intestine, or the pancreas are often summarized as gastroenteropancreatic NET (GEP-NET).^[1]^

In general, different items, predominantly their organ of origin, morphological features, and their grading, classify NET.^[4–6]^ Grading (G1–G3) is deducted from a positive immunohistochemistry (IHC) staining for the proliferation marker Ki67. Other important IHC markers in NET are chromogranin and synaptophysin as well as other markers to determine the originating organ, which is important for the choice of treatment.^[7]^ The WHO classification for NET was changed in 2017, which, among others, harmonized different systems used for small intestinal NET (siNET) and pancreatic NET (pNET) as well as changed the threshold for G1 NET from Ki67 <2% to <3%.^[5]^ Most NET are G1 tumors, which are slowly growing and in a large proportion of patients not detected before a metastasized stage. Besides, patients often do not show any deterioration of their general condition and therefore do not seek medical advice in early stages.^[8–10]^ If patients show symptoms, these are often unspecific and misleading, which might delay diagnosis in many patients.^[4]^ About 20% of all patients experience carcinoid syndrome, which consists of tachycardia, flushing, and diarrhea, either alone or in combination.^[11,12]^ However, these symptoms are not restricted to NET and do not always trigger further targeted diagnostic measures. Sometimes, patients and treating physicians misinterpret them as, for example, symptoms of menopause in women, irritable bowel syndrome, or to be psychosomatic.^[9]^ As a result, patients are reported to often experience a long time from onset of first symptoms to definite diagnosis, which might influence patients’ survival.^[1,9]^

The aim of our study was to analyze, in a large cohort of patients with GEP-NET from a university clinic, the patients’ pre-therapeutic pathways from onset of first symptoms to diagnosis, taking into account NET-specific and unspecific symptoms, and diagnostic measures that detected NET.

## 2 Patients and methods

### 2.1 Study design and ethics

The present retrospective, single center study was performed to investigate the time and pathway to diagnosis for patients with GEP-NET. The study was approved by the institutional review board (internal reference numbers 319/16 and SGI-1-2019) of the University Hospital Frankfurt. Informed consent to participate in the tumor documentation registry was obtained from all patients alive. The informed consent explicitly includes a passage about scientific use. Inclusion criteria of the study were diagnosis with GEP-NET and age ≥18 years. NET with unknown primary tumor were also included.

### 2.2 Patient data

The study database was based on the local electronic hospital charts and was transferred to the local tumor documentation system (Giessener Tumordokumentationssystem). A specific NET dataset was designed and used for documentation of all patients. The dataset included epidemiological and clinical data and is explained in detail in Table S1, Supplemental Digital Content. NET-specific symptoms were defined as diarrhea, abdominal pain, flushing, tachycardia, and carcinoid syndrome (if mentioned specifically without further information), although certainly an overlap between NET specific and non-specific symptoms has to be assumed. Data closure and end of follow-up was February 11, 2019. Table S2, Supplemental Digital Content contains the anonymized date set.

### 2.3 Statistical analyses

Statistical analyses were performed according to international standards and have been described by us and others before.^[12]^ Categorical variables were described in frequencies and percentages. Continuous variables were represented as a median, IQR, and its range. Continuous variables were compared using the Wilcoxon–Mann–Whitney *U* test. Contingency table was analyzed by chi-square or Fisher exact test, as appropriate. All tests were 2-sided and *P* values ≤.05 were considered statistically significant. Analysis was done using International Business Machines Corporation (IBM) Statistical Package for the Social Sciences (SPSS) for Windows (version 22.0; IBM, Chicago, IL), BiAS (version 11, Frankfurt, Germany), and R (R Core Team (2021). R: A language and environment for statistical computing. R Foundation for Statistical Computing, Vienna, Austria).

## 3 Results

### 3.1 Demographics

**Figure 1:**
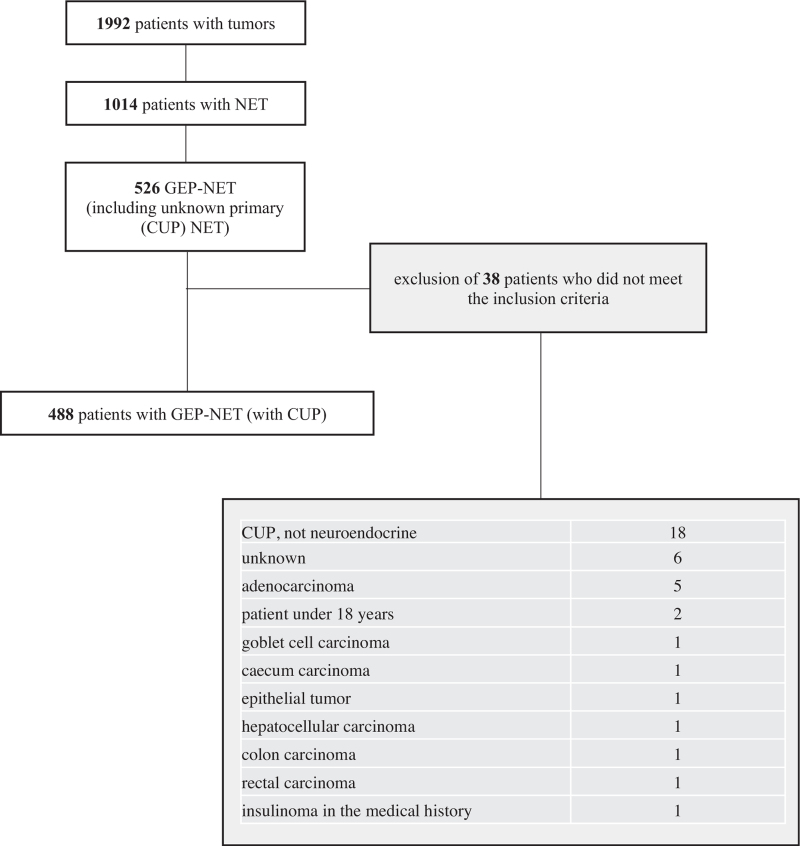
CONSORT diagram. In- and exclusion of patients from the database and generation of the study population. NET = neuroendocrine tumors, GEP-NET = gastroenteropancreatic NET.

486/1030 NET patients in the database fulfilled the inclusion criteria and were included in the study. Further details on patients’ characteristics are lined out in Figure 1. 2/486 patients had 2 different NET tumors. Hence, 488 GEP-NET tumors were detected and counted for further analyses. Age at first diagnosis (478/486, 8 unknown) was 18 to 95 years (median, 59 years). 52.9% of all GEP-NET patients were male, 396/486 alive at database closure. Primary tumor localization is shown in Table 1. The majority of tumors were either pNET (143/488 tumors; 29.3%) or siNET (145/488 tumors, 29.7%).

**Table 1:** Distribution of tumor localization. Number and percentage of primaries at the respective localizations as well as ICD-10 code.

Tumor localization	ICD-10 code	Number of tumors	Percentage
Small intestine	C17	145	29.7%
Pancreas	C25	143	29.3%
Unknown primary (CUP)	C80	53	10.9%
Colon	C18	49	10.0%
Stomach	C16	45	9.2%
Rectum	C20	27	5.5%
Digestive organs not further specified	C26	10	2.0%
Esophagus	C15	5	1.0%
Bile ducts	C24	4	0.8%
Liver	C22	3	0.6%
Anus/anal canal	C21	2	0.4%
Gallbladder	C23	1	0.2%
Peritoneum	C48	1	0.2%

### 3.2 Histology

**Figure 2:**
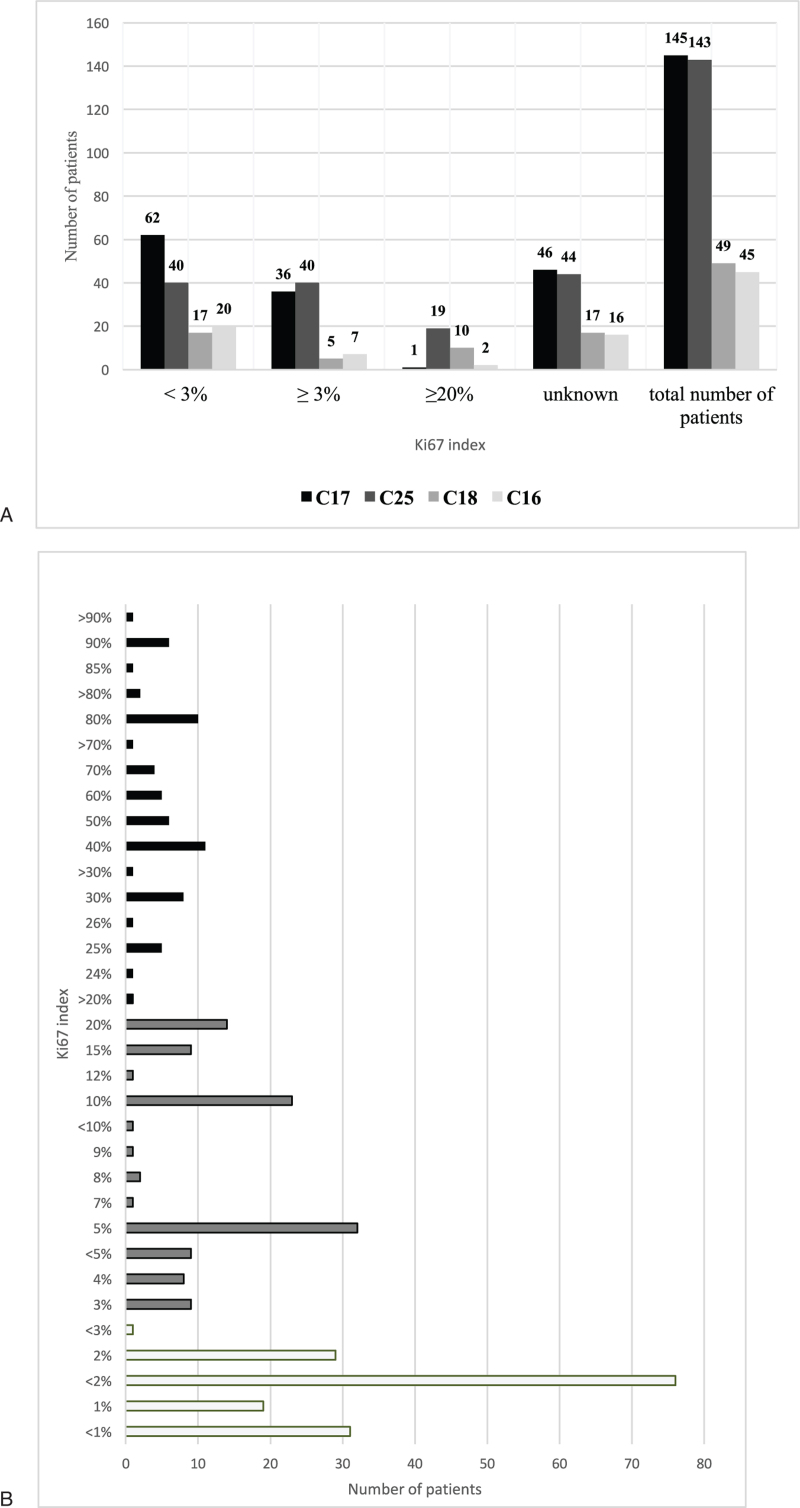
(A) Ki67 index in relation to tumor localization. Number of patients with respective Ki67 indices according to the primary tumor localization. Code numbers refer to the respective ICD classification (ICD-10-GM, version 2021, German modification). C17: small intestinal (si)NET; C25: pancreatic (p)NET; C18: colon NET; C16: gastric NET. (B) Number of patients per reported Ki67 index. Different colors represent different gradings. Light grey: G1 (<3%); grey: G2 (<20%); black: G3 (>20%).

Analysis of histology revealed that 216/488 (44.3%) NET were originally graded G1, 115/488 (23.6%) G2, and 85/488 (17.4%) G3, respectively. However, a detailed review of the Ki67 indices according to the recent WHO classification showed that 155/330 evaluable patients (47.0%) had a Ki67 <3%, 111/330 (33.6%) ≥3% and <20%, and 64/330 (19.4%) ≥20%, respectively. Figure 2A shows the most frequent localizations and Figure 2B the overall distribution of proliferation staining results. IHC results for chromogranin A and synaptophysin are shown in Table 2 and reveal that in less than half of all specimens an IHC was performed at initial diagnosis.

**Table 2:** Immunohistochemistry staining.

	Number of tumors		
	Positive	Negative	Missing
Chromogranin A	212	25	251
Synaptophysin	217	12	259
Both stainings	212	4	247

Number of positive and negative samples for the respective stainings as well as tumors with missing information.

### 3.3 Pathway

**Figure 3:**
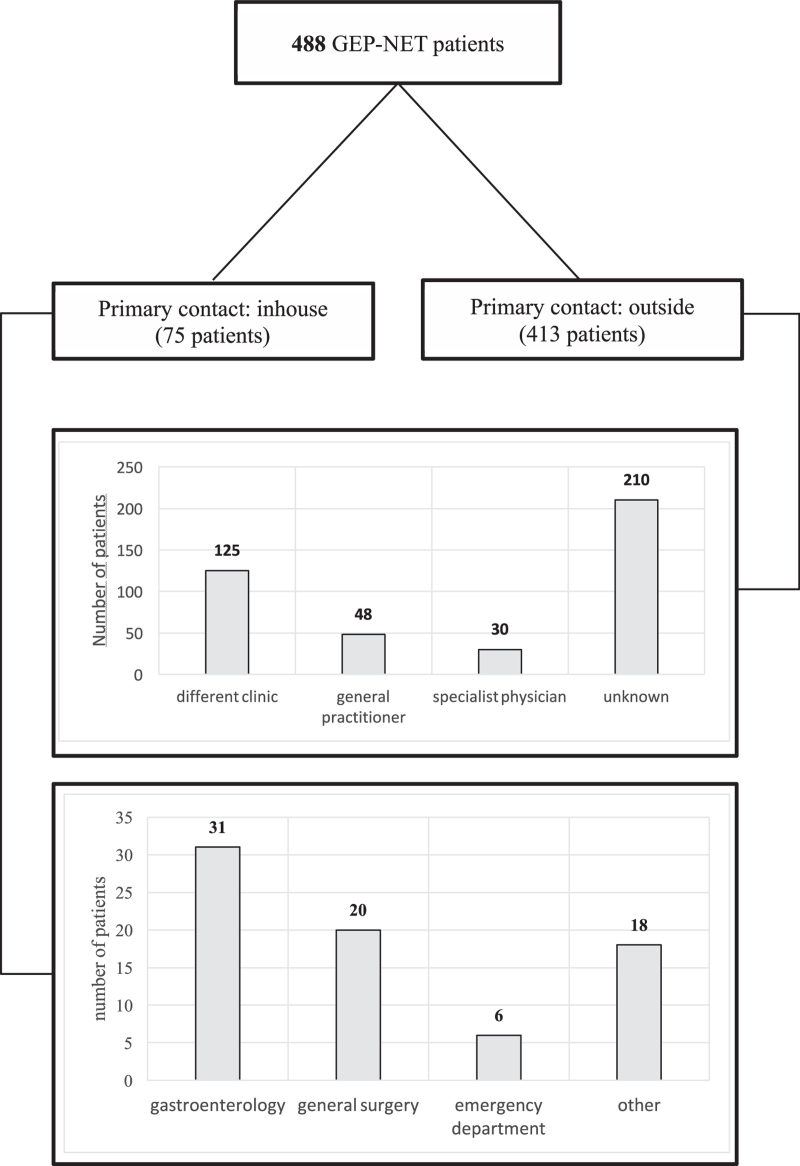
Pre-diagnostic pathway of GEP-NET patients. Primary diagnosis inhouse or outside; number of patients in specific subgroups. GEP-NET = gastroenteropancreatic NET.

Primary contact of the patients was in 75/488 tumors our clinic (15.4%), the remainder of the patients (413/488, 84.6%) were transferred from either another clinic (125/413 patients, 30.3%), a general practitioner or family doctor (48/413, 11.6%) or other physicians with a private practice outside the clinic (30/413, 7.3%). For 210/413 (50.8%) patients, the referring instance was unknown. When patients were diagnosed in our clinic, it was mostly either in the departments of general surgery (20/75, 26.7%) or gastroenterology (31/75, 41.3%) (Fig. 3).

### 3.4 Clinical symptoms

**Figure 4:**
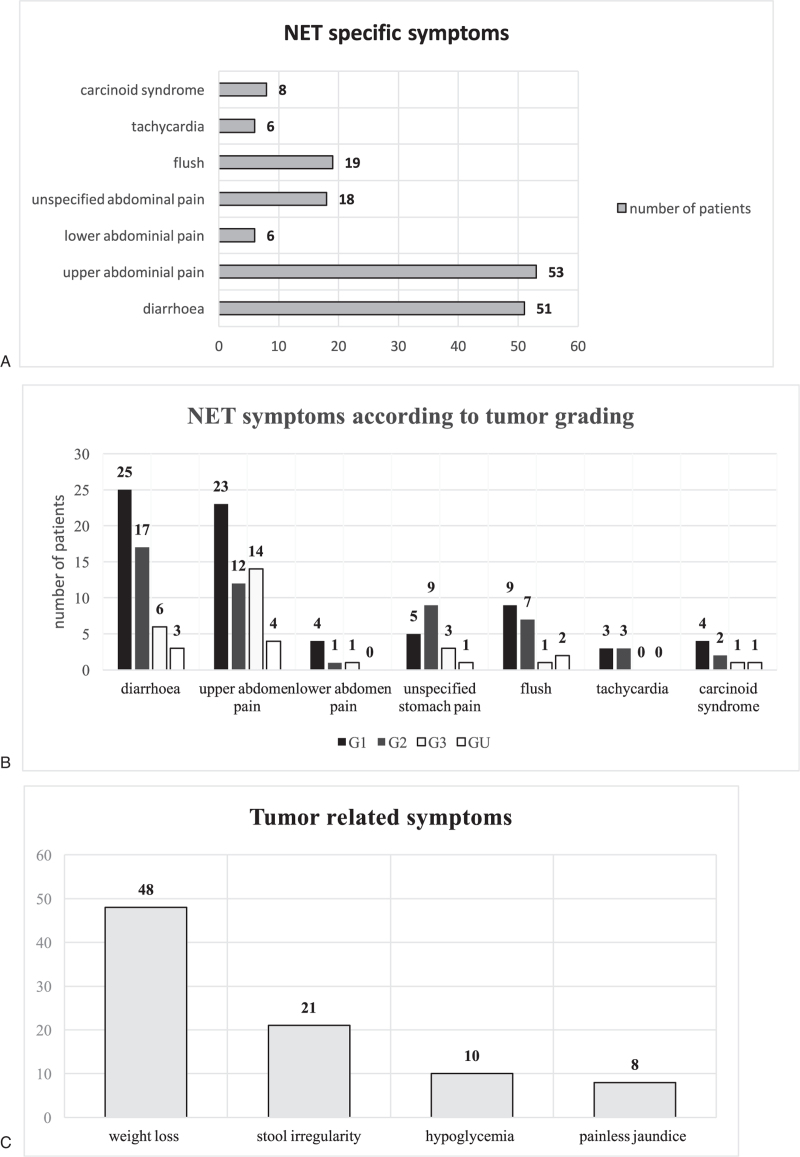
(A) NET specific symptoms. Number of patients with NET specific symptoms. (B) NET symptoms according to tumor grading. Number of patients with respective symptoms; black bars: G1; dark grey bars: G2; light grey bars: G3; white bars: unknown grading. (C) Tumor specific symptoms. Number of patients with tumor related symptoms. NET = neuroendocrine tumors.

For 303/488 tumors, there were sufficient information on tumor associated symptoms noted in the files. 128/303 patients (42.2%) of patients showed NET-specific symptoms according to this definition. 28/128 (21.9%) patients had more than 1 symptom. Details on symptoms and frequency are laid out in Figure 4A and B. 122/488 (25%) patients showed other tumor-specific symptoms, being weight loss in 52/122 (42.6%) patients, stool irregularity in 25/122 (20,5%), hypoglycemia 10/122 (8.2%), and painless jaundice in 8/122 (6.6%) patients, respectively (Fig. 4C). 154/488 (31.6%) of NET were incidental findings (imaging 39.6%, endoscopy 23.4%, surgery for other causes 18.8%, appendectomy 15.6%).

### 3.5 Time to diagnosis

**Figure 5:**
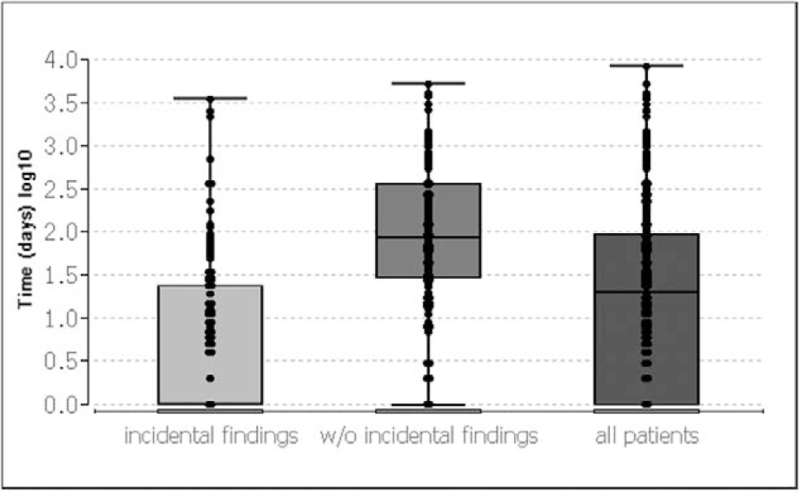
Time to diagnosis. Box plot of all patients with known time to diagnosis across all GEP-NET entities; subgroups: (1) all patients, (2) patients with, and (3) without incidental findings; time to diagnosis in days (log10). IQR, min/max, and outliers are shown. Note the left-skewed data. GEP-NET = gastroenteropancreatic NET.

**Figure 6:**
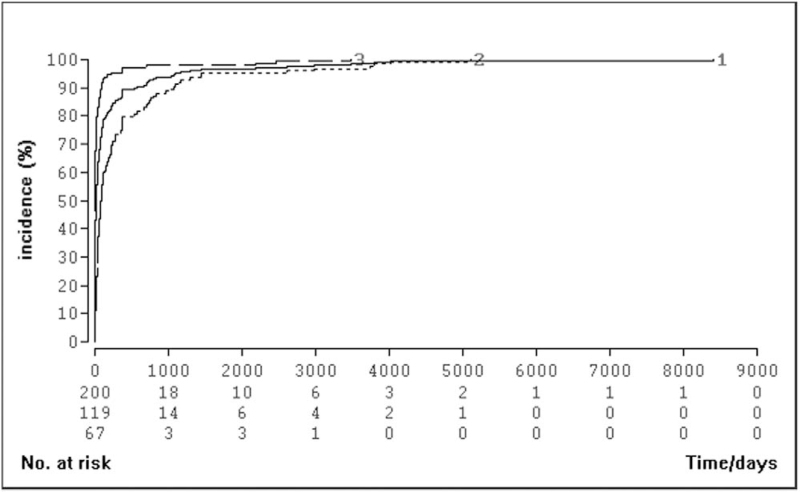
Cumulative frequency of NET diagnoses over time. Cumulative incidence of NET patients over time. Group 1: all patients; group 2: without incidental findings; group 3: incidental findings. NET = neuroendocrine tumors.

For 296 patients, time from first tumor manifestation to definitive diagnosis was known. First tumor manifestation was defined as diagnosis by imaging, onset of first symptoms (NET specific or unspecific) or an incidental finding during surgery or endoscopy. In GEP-NET patients across all entities, median time to diagnosis was 19.5 days (95% CI: 12–28; IQR: 0–93; range: 0–8411 days) for all patients (n = 296), 83.5 days (95% CI: 61–120; IQR: 29–362; range: 0–5113 days) for patients without incidental findings (n = 124), and 0 days (95% CI: 0–5; IQR: 0–24, range: 0–3482 days) for patients whose NET was detected as an incidental finding during another procedure or diagnostic measure (n = 148) (Fig. 5). In 56/296 patients (18.9%), time to diagnosis was ≥180 days and in 40/296 (13.5%) ≥365 days (Fig. 6).

### 3.6 Metastatic patients

In their course of disease, 279/488 tumors metastasized. 222/279 (79.6%) patients had distant metastases at initial diagnosis (187/222 [84.2%] liver metastases). Of the 296 patients for whom information about onset of symptoms were available, 120 (40.5%) had metastases at initial diagnosis. Metastatic patients were not significantly more or less often detected as incidental findings then were non-metastatic patients (at baseline; *P* = .061), which means that metastatic patients were not detected more often because of their symptoms (Table 3).

**Table 3:** Metastatic patients with or without incidental findings.

	Incidental finding: yes	Incidental finding: no	Incidental finding: unknown	∑
Metastasis: yes	49 (32%)	66 (48%)	107 (54%)	222
Metastasis: no	105 (68%)	71 (52%)	90 (46%)	266
∑	154 (100%)	137 (100%)	197 (100%)	488

Contingency table; X -squared = 3.5062, df = 1, *P* value = .061.

**Figure 7:**
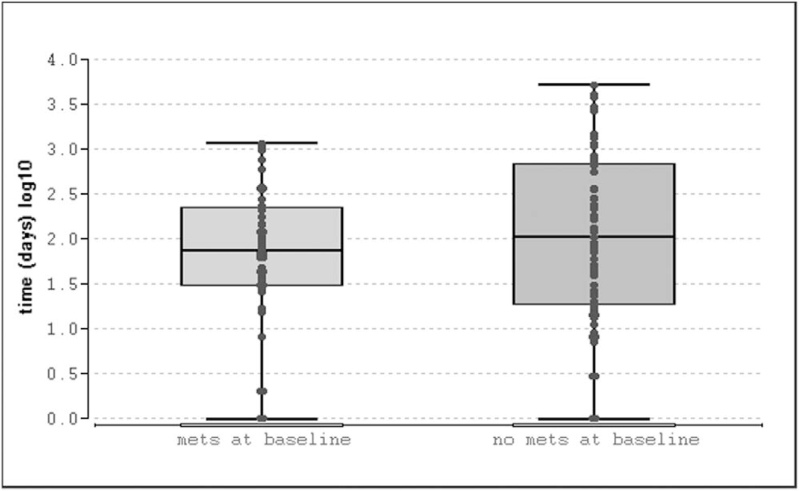
Time to diagnosis in metastatic patients vs non-metastatic patients. (1) Metastases at initial diagnosis and (2) patients without metastases at initial diagnosis; time to diagnosis in days (log10). IQR, min/max, and outliers are shown. Note the left-skewed data.

When excluding patients with incidental findings, time to diagnosis in patients without distant metastases (n = 65) at initial diagnosis vs patients with metastases (n = 59) was not significantly longer (median: 105 days, 95% CI: 46–234, IQR 19–687, vs median: 73 days, 95% CI: 48–109; IQR 31–227, *P* = .42) in patients with metastases (Fig. 7).

## 4 Discussion

NET are a rare disease and diagnosis is anecdotally reported to be delayed sometimes by years. We analyzed a large single center dataset to further clarify symptoms before diagnosis of the tumor and the period until the NET is diagnosed.

Across all entities, median time to diagnosis in our left-skewed data was only 19 days with a wide range. A delay in diagnosis of more than half a year occurred in only about one fifth of our evaluable patients, and 95% of all patients are diagnosed within 5 years. Hence, our data indicate a shorter interval between first tumor manifestation and diagnosis as reported in the literature, regardless of the presence of metastases at initial diagnosis. Incidental findings occurred more often in patients without metastases; in other words, metastatic patients were more frequently diagnosed because of their symptoms.

Assessing time to diagnosis in patients, in general, is difficult: Some tumors are diagnosed as incidental findings, for example, during surgery, endoscopy, or imaging for other causes, which leads to a time to diagnosis of 0 up to a few days. Other patients retrospectively report symptoms when their medical history is taken at initial diagnosis, and these might be NET specific, such as flushing or diarrhea, or unspecific, such as weight loss. However, even NET “specific” symptoms might arise from other conditions and diseases, or patients might have a varying awareness for their symptoms. Hence, determining the real time from onset of first symptoms to definite diagnosis seems difficult and biased.

One large global survey on patient-reported outcomes was undertaken in 2014 and found a mean time to diagnosis of 59 months for US GEP-NET patients^[13]^ and 52 months for the global population^[10]^ across all entities. For 29% of all patients of the global population, time to diagnosis was longer than 5 years. Another survey found a mean time from symptom onset to diagnosis of 36 months for siNET and 24 months for pNETs.^[14,15]^ Both datasets are based on patient-reported outcomes and did not include a review of the patients’ files. Also, there is no detailed information as to whether the time distribution was skewed towards shorter or longer periods between symptom onset and definite diagnosis. If longer periods to diagnosis are outliers, median instead of mean time to diagnosis might be a more realistic parameter.

We took a different approach by reviewing the patients’ files and collecting all information that was available at primary diagnosis. Thereby, we were able to better exclude bias due to a false or incomplete recall by the patient. However, we had complete information only for about 60% of our patients. Also, we divided the cohort into different sub-cohorts by type of primary diagnosis, primary tumor site, and whether the patient was symptomatic or not.

Demographics and distribution of histology in our cohort were as expected from the literature^[16–18]^; therefore, we assume that we analyzed a representative cohort and that the results could be transferrable to other tertiary centers.

Considering the difficulties in diagnosing the tumor early, we also tried to better understand the pathway to diagnosis for our patients. We were not able to get information on the number of contacts with a physician or health care provider before diagnosis as reported by Singh et al^[10]^: however, we found that patients see different referring instances and clinics before a definite diagnosis, which underscores the importance of a family doctor or GP as a coordinating instance. Also, since a majority of patients shows liver metastasis at initial diagnosis, routine ultrasound examinations might facilitate early diagnosis.

Limitations of our study are the retrospective design and the large time span with partly incomplete datasets.

## 5 Conclusion

Taken together, we were not able to confirm the reported massive delay in diagnosis for the majority of patients. Although it would be desirable to detect tumors in a not metastasized stage in order to be able to perform surgery, there are only rarely “red flag” symptoms such as ileus or massive carcinoid syndrome that should have prompted further diagnostic measures. Health care providers should consider NET as a differential diagnosis in patients with unknown findings in endoscopy or imaging. In patients with carcinoid syndrome, which occurs only in metastasized patients, an abdominal ultrasound could detect liver metastases early.

## Acknowledgment

The authors would like to thank Mr Gerald Koch, MBA, for critically reading the manuscript and technical advice.

## Author contributions

**Conceptualization:** Christine Koch, Jörg Bojunga.

**Data curation:** Christine Koch, Esra Koca, Gabriele Husmann.

**Formal analysis:** Christine Koch, Esra Koca.

**Funding acquisition:** Jörg Bojunga.

**Investigation:** Christine Koch, Esra Koca.

**Methodology:** Natalie Filmann.

**Project administration:** Christine Koch, Jörg Bojunga.

**Resources:** Gabriele Husmann, Jörg Bojunga.

**Supervision:** Christine Koch, Natalie Filmann, Gabriele Husmann, Jörg Bojunga.

**Validation:** Christine Koch, Esra Koca, Natalie Filmann.

**Visualization:** Christine Koch, Esra Koca.

**Writing – original draft:** Christine Koch, Esra Koca.

**Writing – review & editing:** Christine Koch, Esra Koca, Natalie Filmann, Gabriele Husmann, Jörg Bojunga.

## Supplementary Material

Supplemental Digital Content

## Supplementary Material

Supplemental Digital Content
